# Influence of demographic and socio-economic factors in choosing hospitalist careers among US medical students

**DOI:** 10.1186/s12909-022-03792-y

**Published:** 2022-10-25

**Authors:** Jean-Sebastien Rachoin, M. Olguta Vilceanu, Natali Franzblau, Sabrina Gordon, Krystal Hunter, Elizabeth Cerceo

**Affiliations:** 1 Division of Hospital Medicine, Department of Medicine, Cooper University Healthcare, Cooper Medical School of Rowan University, Camden, NJ United States of America; 2 Department of Public Relations and Advertising, Rowan University, Glassboro, NJ United States of America; 3 Department of Obstetrics and Gynecology, Cooper University Healthcare, Cooper Medical School of Rowan University, Camden, NJ United States of America; 4 Department of Medicine, Cooper University Healthcare, New Jersey, Camden, United States of America; 5 Cooper Research Institute, Cooper Medical School of Rowan University, Camden, NJ United States of America

**Keywords:** Medical students, Hospitalists, Career choice, Demographic factors

## Abstract

**Background:**

The subspecialty of Hospital Medicine (HM) has grown rapidly since the mid-1990s. Diversity and inclusion are often studied in the context of healthcare equity and leadership. However, little is known about the factors potentially associated with choosing this career path among US medical students.

**Methods:**

We analyzed the results of the Annual Association of American Medical Colleges Survey administered to Graduating medical students from US medical schools from 2018 to 2020.

**Results:**

We analyzed 46,614 questionnaires. 19.3% of respondents (N = 8,977) intended to work as a Hospital Medicine [HM] (unchanged from 2018 to 2020), mostly combined with specialties in Internal medicine (31.5%), Pediatrics (14.6%), and Surgery (9.1%). Students interested in HM were significantly more likely to identify as female, sexual orientation minorities (Lesbian/Gay or Bisexual), Asian or Black/African-American, or Hispanic. Role models and the ability to do a fellowship were strong factors in choosing HM, as was higher median total debt ($170,000 vs. $155,000). Interest in higher salary and work/life balance negatively impacted the likelihood of choosing HM. There were significant differences between students who chose IM/HM and Pediatrics/HM.

**Conclusion:**

About one in five US medical students is interested in HM. The probability of choosing future HM careers is higher for students who identify as sexual or racial minorities, with a higher amount of debt, planning to enter a loan forgiveness program, or are interested in doing a fellowship.

## Background:

Hospital medicine [HM] has grown rapidly since the model was described in the United States in the mid-nineties [1]. From fewer than 1,000 in 1996, this new specialty grew to 38,000 in 2012 and more than 50,000 in 2016 [2, 3]. Hospitalists are physicians who specialize in taking care of patients only in the hospital setting. They are generalists that train in either Internal medicine [IM], family medicine (adult hospitalists), or pediatrics (pediatric hospitalists), treat all patients in the hospital, and do not restrict their practice to a specific area or field. Hospitalists are considered primary services in the hospital and when they care for patients may request help from consultants. Subspecialists (such as Cardiologists or oncologists) are not considered hospitalists as they focus on a specific organ system and provide (mostly) consultative services in the hospital setting. Some of the reasons that help explain this growth include economic and efficiency trends, availability of a large internal medicine inpatient trained workforce, and the focus on quality, patient safety and improvement of healthcare systems [3]. Hospital medicine has expanded from its initial focus on care for adult medical inpatients to caring for pediatric, neurological, and surgical or obstetric patients [4–7].

Currently, it is estimated that 9–10% of IM graduates choose careers in hospital medicine [8] and most of them decide in their last year of residency. As per Dr. Chopra (Chief of Hospital Medicine at University of Michigan Health System), future hospitalists tend to be interested in practicing in academic hospitals, or “curious as to what it means to be a generalist in the hospital; and those who have already matched into fellowship but have a gap year before beginning training. ” [9].

With Medicare programs increasing financial pressure on hospitals, inpatient care is facing the challenge of increasing the quality of service and treatment, while reducing the duration of hospital stay. The healthcare system is also facing pressure to improve communication and care, as reflected in the Hospital Consumer Assessment of Healthcare Providers and Systems [HCAHPS]. Hospitalists are at the forefront of addressing these challenges. Recent research pointed out that both quality and satisfaction with service improve when patients face fewer obstacles in communicating with their providers–be it language, gender, race/ethnicity, and other cultural markers [10, 11]. The changing demographics of patients are also challenging the long-standing history of medical solutions and treatments, being developed primarily for white patients who used to be able to afford or access them. [12] Disparities of care, today, are increasingly the result of communication and access to comprehensible information that feels relatable for patients from diverse backgrounds and perspectives.

While the choice to pursue a career in hospitalist medicine is made during residency training, the interest starts before graduating from medical school. Analyzing who shows interest during the clinical years can help better direct schools and residency programs in their education and recruitment to promote diversity in the field.

We designed a study whose purpose is to: quantify how frequently are interested in pursuing a career in hospital medicine, and explore socio-demographic factors and identify characteristics associated with a higher likelihood of choosing hospital medicine in general, and adult IM or pediatric hospitalist careers in particular.

## Methods

### Participants and survey

The Association of American Medical Colleges (AAMC) administers an annual survey to all fourth-year students in 153 US medical schools, called the Graduation Questionnaire (GQ). Response rate during the study period included completed questionnaires from over 81.6% of the US graduates. The survey “is an important tool for medical schools to use in program evaluation and to improve the medical student experience” (aamc.org) ”[13]. It includes questions regarding career intentions, indebtedness and general medical education.

This questionnaire is a very important tool used by the AAMC to help improve medical student’s experiences and identify trends or areas that need improvement. It has already been used in other research projects and is considered very reliable [14].

### Student sample and inclusion criteria

AAMC data from GQ administered during 2018–2020 was acquired in de-identified format. All the analyses and results were reviewed and approved by the AAMC. The Rowan University IRB approved the exempt status for research using this de-identified data.

The following question was used as a filter: “Do you plan, at some point in your career, to work as a hospitalist (i.e., full-time care of hospitalized patients)?” The following answer options were provided: yes, unsure, and no.

We included students who responded to the hospitalist question with any answer. We excluded students who answered incorrectly the gender question or did not answer the hospitalist question. The total sample included consisted of 46,614 students. (94.1% of original dataset).

### Independent variables

The following self-reported demographic variables were used for this study: age range, gender, sexual orientation and race. While the GQ survey does not collected race responses itself, it is able to link with other AAMC applications. For the race variable, respondents who reported more than one race were considered “multiracial.” With respect to the socio-economic factors, the following variables were considered: the amount of debt by graduation (total debt), plans to apply for loan forgiveness, and factors influencing students’ choice of specialty. Answers to the factor questions were grouped as follows: “minor or no influence” and “moderate or strong influence.”

### Statistical analysis

We presented continuous data as median, 25–75% interquartile range, and categorical variables as percentages. Interdependence of factors regarding the choice to work as a hospitalist was determined using univariate analysis with Chi-square and Mann-Whitney tests, and multivariable regression analysis. We used forward conditional methodology and considered variables to have a significant association with the outcome of interest if p < 0.05. All the variables were categorical, with the exception of the debt amount. All analyses were done using SPSS, IBM 28.0 software (Chicago, IL, USA).

## Results

Of the students who answered the question about intending to pursue a career in Hospital Medicine (HM) in the 2018–2020 study period, about 19.3% (N = 8,977) were interested, and 34.3% were unsure (%, N = 15,972) or not interested (46.5%, N = 21,665). The proportion of students interested in working as hospitalists was relatively stable for the three years in the study period: 19.4% in 2018, 19% in 2019, and 19.4% in 2020 (p = 0.627).

### Demographic characteristics of students interested in hospital medicine

Compared to students who answered “unsure” or “no”, students who answered “yes” were more likely to be 29 or younger, reflecting the traditional student age range, or female (52.6% vs. 49.9%, p < 0.001); bisexual (3.9% vs. 3.1%) or gay/lesbian (4.5% vs. 3.8%)--rather than heterosexual (89.4% vs. 91%) (p < 0.001); and Hispanic (7.4% vs. 4.9%), African American (6.3%vs 5.3%), or Asian (24.2 vs. 20.5%)--rather than White (51.4% vs. 58.7%) (p < 0.001). (Table 1)

**Table 1:** Demographic variables

	HM No/Unsure	HM Yes	Total	P value
**Total**	37,637 (80.7%)	8,977 (19.3%)	46,614	
**Age**				
< 24	119 (0.3%)	32 (0.4%)	151 (0.3%)	
24–26	14,855 (39.5%)	3,730 (41.6%)	18,585 (39.9%)	
27–29	16,024 (42.6%)	3,773 (42%)	19,797 (42.5%)	< 0.001
30–32	4,493 (11.9%)	960 (10.7%)	5,453 (11.7%)	
33 or older	2,146 (5.7%)	482 (5.4%)	2,628 (5.6%)	
**Gender: Female**	18,771 (49.9%)	4719 (52.6%)	23,490 (50.4%)	< 0.001
**Sexual Orientation**				
Bisexual	1,150 (3.1%)	349 (3.9%)	1,499 (3.2%)	
Gay or Lesbian	1,439 (3.8%)	407 (4.5%)	1,846 (4%)	< 0.001
Heterosexual No answer	34,260 (91%) 788(2.1%)	8,028 (89.4%) 193(2.1%)	42,288 (90.7%) 981(2.1%)	
Race*				
Asian	7,700 (20.5%)	2,172 (24.2%)	9,872 (21.2%)	
Black	1,998 (5.3%)	566 (6.3%)	2,564 (5.5%)	
Hispanics	1,842 (4.9%)	660 (7.4%)	2,502 (5.4%)	< 0.001
Multiracial	3,182 (8.5%)	742 (8.3%)	3,924 (8.4%)	
Not disclosed	101 (0.3%)	14 (0.2%)	115 (0.2%)	
Other**	713(1.9%)	212(2.4%)	925 (2%)	
White	22,101 (58.7%)	4,611 (51.4%)	26,712 (57.3%)	
**Specialties**				
Internal Medicine	6,038 (16%)	2,827(31.5%)	8,865 (19%)	
Pediatrics	3,316(8.8%)	1,313 (14.6%)	4,629 (9.9%)	< 0.001
Surgery	2,066 (5.5%)	814 (9.1%)	2,880 (6.2%)	

HM: Students who chose Hospital medicine

*Numbers include only individuals who indicated a single race; for individuals who selected two or more races, see “Multiracial.”

**Includes “Other, Pacific Islander, Native American/Alaskan

### Socio-economic factors influencing choice of hospitalist careers

When asked what influenced their career choice, future hospitalists (compared to those who were unsure or not interested) expressed strong or moderate importance for the following factors: role models (82.4% vs. 80.5%, p < 0.001), ability to do a fellowship (69.3% vs. 60.7%, p < 0.001), and length of training (44.1% vs. 42.8%, P = 0.007). Other factors such as competitiveness of the specialty or family expectations exerted no influence on the hospitalist career choice.

Conversely, moderate or strong influence of the following factors were associated with lower likelihood of interest in a hospitalist career: educational debt (20.6% vs. 21.6%, p = 0.012), interest in higher salary (42.4% vs. 46.7%, p < 0.001), influence of their future family plans (54.6% vs. 57.0% p < 0.001), and work/life balance (73.5% vs. 76.8% p < 0.001). Two other factors presented a negative correlation, between moderate or strong influence, and interest in hospitalist careers: importance of the specialty-matching respondents’ personality, interest, and skills; and the content of the specialty: there was a near-unanimous response on both questions. (Table 2)

**Table 2:** Influencing factors for HM Career Intention (Moderate & Strong Influence)

Factors influencing	HM Yes N = 8,977 (19.3%)	HM No/Unsure N = 37,637 (80.7%)	All students N = 46,614 (100%)	P value HM yes vs. No/ unsure*	IM/HM N = 2,827	Pediatrics/HM N = 1,313	P value IM/HM vs. P/HM**
Competitiveness of specialty	3,449 (38.4%)	14,561 (38.7%)	18,010 (38.6%)	0.647	1,160 (41.2%)	301 (22.9%)	< 0.001
Level of educational debt	1,847 (20.6%)	8,203 (21.8%)	10,050 (21.6%)	0.012	688 (24.3%)	128 (9.7%)	< 0.001
Role models	7,399 (82.4%)	30,113(80%)	37,512 (80.5%)	< 0.001	2,373 (83.9%)	1,080 (82.3%)	0.175
Fellowship options	6,222 (69.3%)	22,066(58.6%)	28,288 (60.7%)	< 0.001	2,256 (79.8%)	949 (72.3%)	< 0.001
Income Expectations	3,802 (42.4%)	17,958 (47.7%)	21,760 (46.7%)	< 0.001	1,381 (48.9%)	218 (16.6%)	< 0.001
Length of residency training	3,957 (44.1%)	15,994 (42.5%)	19,951(42.8%)	0.007	1,647 (58.3%)	480 (36.6%)	< 0.001
Family expectations	2,680 (29.9%)	11,016 (29.9%)	13,696 (29.4%)	0.279	917 (32.4%)	347 (26.4%)	< 0.001
My future family plans	4,900 (54.6%)	21,647 (57.5%)	26,547 (57.0%)	< 0.001	1,677 (59.3%)	756 (57.6%)	0.289
Work/Life Balance	6,600 (73.5%)	29,215 (77.6%)	35,815 (76.8%)	< 0.001	2,331(82.5%)	1,048 (79.8%)	0.041
Fit with personality, interests, and skills	8,762(97.6%)	36,930 (98.1%)	45,692 (98.0%)	0.002	2,742 (97.0%)	1,296 (98.7%)	< 0.001
Content of specialty	8,716 (97.1%)	36,744 (97.6%)	45,460 (97.5%)	0.004	2,723 (96.3%)	1,280 (97.5%)	0.051
Total Debt (in 1,000 $)	170 [10–250]	155[0-242,724]	160 [0-246]	< 0.001	160 [0-247.75]	170 [19.5–250]	0.167
Plan to enter loan forgiveness Prg.	3471 (38.7%)	11,983(31.8%)	15,454 (33.2%)	< 0.001	969(34.3%)	585(44.6%)	< 0.001

HM: Students who chose Hospital medicine, Prg: Program

(*) HM vs. All others

(**) IM/HM vs. Pediatrics/HM

Finally, future hospitalists tended to have less physicians without debt (24% vs. 26.6%, p < 0.001) average higher levels of total debt ($170,000) when compared to those who were unsure or not interested ($155,000), and more interest in entering loan forgiveness programs (38.7% vs. 31.8%, p < 0.001).

### Hospital medicine and specialty choices

Among future hospitalists, the most frequent specialties included Internal Medicine (IM) (31.5%), Pediatrics (14.6%), and Surgery (9.1%). (Table 1)Within each specialty, however, the percent of respondents interested (vs. unsure or not interested) in HM varied, with highest representation in IM/pediatrics (422/857 or 49%), IM (2,827/8,865 or 31.9%) and Pediatrics (1,313/4,629 or 28.4%).

### Hospitalists: internal medicine vs. pediatrics

There were significant differences when we compared data from the students who were interested in Hospital Medicine and chose Internal Medicine, versus Pediatrics as their specialty. For example, IM respondents were more likely to indicate a moderate or strong influence on their career choices for factors such as: competitiveness of specialty (41.2% vs. 23% p < 0.001), more debt (24.3% vs. 9.7% p < 0.001), options for fellowship training (79.8% vs. 72.3%, p < 0.001), salary expectations (48.9% vs. 16.6% p < 0.001), work/life balance (82.5% vs. 79.8%, p = 0.041), length of residency training (58.3% vs. 36.6% p < 0.001), and family expectations (32.4% vs. 26.4% p < 0.001). IM students were also less likely to plan to enter a loan forgiveness program (34.3% vs. 44.6% p < 0.001) than Pediatrics students. There were no differences in factor influence on career choice for questions about mentorship, family planning, content of specialty, or amount of debt. (Table 2)

### Multivariable analysis

We performed a multivariable regression analysis for the outcome: choice of HM. Multivariable analysis determined that minority status in race or sexual orientation, length of residency training, amount of debt, plans to enter a loan forgiveness program, and options for fellowship training were all independently associated with a higher likelihood of interest in HM (Table 3). Factors associated with lower likelihood were salary expectations, work/life balance, personality fit and content of specialty. (Table 3)

**Table 3:** Multivariable regression analysis for the outcome: choice of HM

	OR [95% CI]	P value
Level of Educational Debt	0.87[0.81–0.94]	< 0.001
Salary	0.73[0.68–0.78]	< 0.001
Future family plans	0.91[0.84–0.98]	0.01
Work/Life Balance	0.81[0.75–0.88]	< 0.001
Content	0.78[0.64–0.93]	0.007
Fellowship options	1.71[1.60–1.81]	< 0.001
Length of training	1.23[ 1.15–1.31]	< 0.001
Family expectations	1.14[1.06–1.22]	< 0.001
Entering loan forgiveness	1.3[1.15–1.38]	< 0.001
Debt amount ( /10 K)	1.01[1.23–1.38]	0.001
Asian (vs. White)	1.43[1.33–1.54]	< 0.001
Black (vs. White)	1.32[1.18–1.47]	< 0.001
Hispanic (vs. White)	1.61[1.45–1.8]	< 0.001
Multiracial (vs. single-race)	1.13[1.01–1.25]	0.026
Other (vs. White)	1.42[1.17–1.73]	< 0.001
Bisexual (vs. Het)	1.29[1.11–1.48]	< 0.001
Gay (vs. Het)	1.3[1.13–1.18]	< 0.001

HM: Students who chose Hospital medicine, Het: Heterosexual

## Discussion

Understanding the characteristics of students interested in pursuing HM can support not only effective changes and adaptations in medical education programs, but also identification of key issues relevant to recruitment of hospitalist physicians. We found consistent diversity characteristics among students interested in going into HM in terms of socio-economic background, race, gender, and sexual orientation. Comparing internal medicine- versus pediatric-based future hospitalists revealed relatively similar demographics for the two groups, whereas they held divergent views in some of their career opinions and preferences.

Students interested in HM are significantly more diverse than those who are unsure, or not interested in this career path. Future hospitalists tend to have a higher proportion of minority characteristics, be they racial (multiracial or non-White), gender (women), or sexual orientations (LGB). As the group of students interested in hospitality medicine becomes more reflective of the overall patient population, there is the potential for actual and perceived higher quality of care delivered to increasingly diverse patients.

Issues of student debt across higher education have raised considerable debate. Although the surveys do not necessarily shed light on students’ individual socioeconomic background, it is conceivable that those with greater student debt may come from families with less means to support their education financially. This is notable since the majority of medical students come from more affluent backgrounds: over 50% of medical students are from families in the top-20% for household income [15]. Prioritizing shorter fellowship programs might also be a proxy for financial concerns and a need to acquire financial stability, rather than prolong and increase debt.

When choosing their specialty, students’ psycho-demographics already influence their choices, for example their intent to go into Internal Medicine vs. Pediatrics. When looking at students interested in HM, there were significant differences between IM and pediatric specialties. However, the importance of role models was evident in both HM/IM and HM/Pediatrics groups, confirming that current hospitalists are crucial in students’ professional development. Among students interested in HM, there were significant differences between IM and pediatric specialties. Those expressing interest in IM acknowledged stronger influence by factors such as salary and debt, and were less likely to plan to enter loan forgiveness programs. They also attributed stronger influence to the possibility of doing a fellowship and the competitiveness of the specialty, along length of residency training prior to entering practice. Work/life balance was slightly more important for those leaning toward HM/IM, but was recognized as a moderate or strong factor for most future Hospitalists (about 4 out of 5). Among students interested in med-peds, almost half (49%) wanted to pursue a career in HM.

Students interested in HM are significantly more diverse than those who are unsure or not interested in this career path. Future hospitalists tend to have a higher proportion of minority characteristics, be they racial (multiracial or non-White), gender (women), or sexual orientations (LGB). There are strong trends in choosing HM among students who identify as racial minorities. Additionally, students trained at diverse schools are more comfortable treating patients with a wide variety of racial and ethnic backgrounds, just as patients tend to report higher levels of trust and satisfaction when they share demographic characteristics with their primary care physicians [16, 17]. It is encouraging to find strong trends in choosing HM among students who identify as racial minorities. For example, Black, American Indian, and Alaska Native women are two to three times more likely to die from pregnancy-related causes than white women [18]. However, this is mitigated when patients and physicians have concordant racial backgrounds [19]. When patients enter our hospitals, they want to see staff members and physicians who resemble them and there are better outcomes with greater concordance. Hospital recruitment efforts should thus attempt to engage physicians who mirror the population served for both patient safety and satisfaction reasons and this work can extend into medical school.

Diversity is in itself a laudable goal for programs. Individuals from diverse backgrounds can contribute a wide depth and breadth of perspectives, which allows for more creativity and innovation, but also fosters a more positive working environment and can improve morale among healthcare professionals and patients. Our ability to communicate and care for people from diverse socio-cultural backgrounds, expectations, values, and beliefs define our interaction with patients. Acknowledging the fact that HM attracts students who identify as racial, gender, or sexual orientation minorities allows for better alignment of institutional and programmatic goals with greater physician satisfaction. This has important implications for recruitment and retention. Programs that offer DEI initiatives, value diverse talent acquisition, and align these goals with their mission and vision may be able to more successfully build their body of future Hospitalists and attain greater engagement.

As pragmatic implications, initiatives that reach out early in medical students’ careers might improve their output of future hospitalists. Understanding trends in medical students’ diversity can also inform the creation of pipeline medical programs, at both undergraduate and graduate levels. Recognizing the needs and interests of medical trainees will help programs adapt and maximize recruitment. In graduate medical education (GME), residency programs are creating differentiated tracks among which HM is becoming increasingly popular. Incorporating DEI efforts and cultural competency is integral to the residency curriculum. While a large number of fourth-year medical students (1 in 5) reported being interested in HM, the size of future cohorts of hospitalists might be diminished through the process of completing residency, fellowships, and other training. Medical students are exposed to academic hospitalists for both teaching and mentorship, with hospitalists serving important roles in improving in-patient care and reducing costs through decreasing duration of patient stays [20].

Surveys modelled on the GQ should be administered nationally during residency, when medical trainees are one-step closer to finalizing their career choices. Such research would help improve general knowledge about the practical aspects of working as a hospitalist, possibly in combination with other specialties. At a minimum, findings such as these insights from GQ surveys can inform program design, content, and emphasis and can provide discussion points for individual hospitalist divisions.

Limitations of using a large survey database include the inability to discern nuance in the questions or to delve more deeply into what individual respondents meant. Although the instruments are comprehensive, there may certainly be other factors that are not captured in this survey but that impact students’ decisions. Focus groups and interviews may help create a more well-rounded picture of the factors that inform career decisions. Additionally, as students continue their residency, their patterns of interest may change. Using data from fourth year medical students does not provide certainty that these students will continue to pursue HM. There is a strong need to build data repositories for longitudinal studies that will inform a more complete picture of the characteristics of trainees who will ultimately enter each field. Another area where more research is imperative includes extension of similar surveys into GME.

Future directions should include targeted and detailed research into factors influencing students decisions to pursue specific fields including how clinical exposure impacted career choice. Focus groups and interviews may help create a more well-rounded picture of the factors that inform career decisions. Extending the studies into residencies and beyond into clinical practice can help support the needs and interests of physicians as they navigate an increasingly difficult landscape and one in which burnout is rampant. Aligning interests, practical considerations of the give field, and career potential may be an important component in building resiliency and improving retention. Additional research into optimal training exposure would help improve general knowledge about the practical aspects of working as a hospitalist, possibly in combination with other specialties. At a minimum, findings such as these insights from GQ surveys can inform program design, content, and emphasis and can provide discussion points for individual hospitalist divisions.

Assessing characteristics of trainees across the educational continuum can build pipelines to strengthen and grow hospitalist programs and align interests of students and residents for optimal recruitment and retention, ultimately with a goal to improve patient and physician satisfaction.

## Conclusion

About one in five US medical students are interested in HM. The probability of choosing future HM careers is higher for students who identify as sexual or racial minorities, with a higher amount of debt, planning to enter loan forgiveness program, or interested in doing a fellowship.

## Data Availability

The dataset used for this study was obtained and used with the permission of the American Association of Medical colleges (AAMC) for the sole purpose of this research project. The AAMC reviewed and approved the results and manuscript. The data can be obtained from the AAMC after a written request. We are not permitted to make it available publicly. David Matthew from the AAMC The AAMC can be contacted to obtain the dataset. dmatthew@aamc.org.
